# Rare Presentation of a Rare Tumor: Histiocytic Sarcoma

**DOI:** 10.7759/cureus.7770

**Published:** 2020-04-21

**Authors:** Anil Kumar Reddy Reddivari, Parth Mehta, Usha Sree Janapala

**Affiliations:** 1 Internal Medicine, University of Illinois College of Medicine, Peoria, USA; 2 Internal Medicine, Unity Point Health Methodist Hospital, Peoria, USA; 3 Neurology, University of Illinois College of Medicine, Peoria, USA

**Keywords:** histiocytic, sarcoma, mass

## Abstract

An 84-year-old male, with a large gouty tophus over the left elbow for 20 years, developed a new left elbow area mass about three months ago and presented to the emergency department (ED) with complaints of bleeding from that left elbow area mass after a small blunt injury. Initially diagnosed as olecranon bursitis, it had the worsening size and atypical growth associated with persistent leukocytosis. After being evaluated by multiple specialties, including infectious diseases, orthopedic surgery, and rheumatology, it was decided to do a biopsy, which resulted in the diagnosis of histiocytic sarcoma.

## Introduction

Histiocytic sarcoma is a very rare disease belonging to histiocytic disorders that originate from histiocytic cells that are of hematopoietic origin. Histiocytic sarcoma may occur as a sporadic extranodal neoplasm or may be a complication of hematologic malignancies such as follicular lymphoma or acute lymphoblastic leukemia. Histiocytic sarcoma (HS) is an aggressive neoplasm with a rapidly progressive course. With only a few hundred cases reported in the medical literature, HS contributes to less than 1% of all hematopoietic malignancies [1].

Clinical presentation is mostly a neoplastic mass, extranodal in location, commonly involving the skin. Other sites involved are the spleen and gastrointestinal tract. Associated signs and symptoms are nonspecific systemic symptoms such as fever, weight loss, anorexia, fatigue, hepatosplenomegaly, lymphadenopathy, rash, and pancytopenia. Diagnosis is made by the excisional or incisional biopsy of neoplastic tissue. Treatment options include local surgery, radiotherapy, and chemotherapy, depending on the stage of the disease. Being so rare, diagnosis is usually delayed and the overall prognosis of histiocytic sarcoma is very poor.

Here we submit a case report of a rare presentation of a very rare disease.

## Case presentation

An 84-year-old man with a past medical history of gouty arthritis, hypertension, chronic kidney disease stage III, cardiomyopathy, and atrial fibrillation presented to the emergency room with complaints of bleeding from the left elbow area. The patient had developed a mass about three months ago over the left elbow and was evaluated by his orthopedic surgeon, who diagnosed it as olecranon bursitis. Bleeding started after his elbow bumped into his car door. Vital signs at the time of presentation: blood pressure 99/60, pulse 100 per min, temperature 36.6 °C, respiratory rate 18 per min, and saturation 98%.

**Figure 1:**
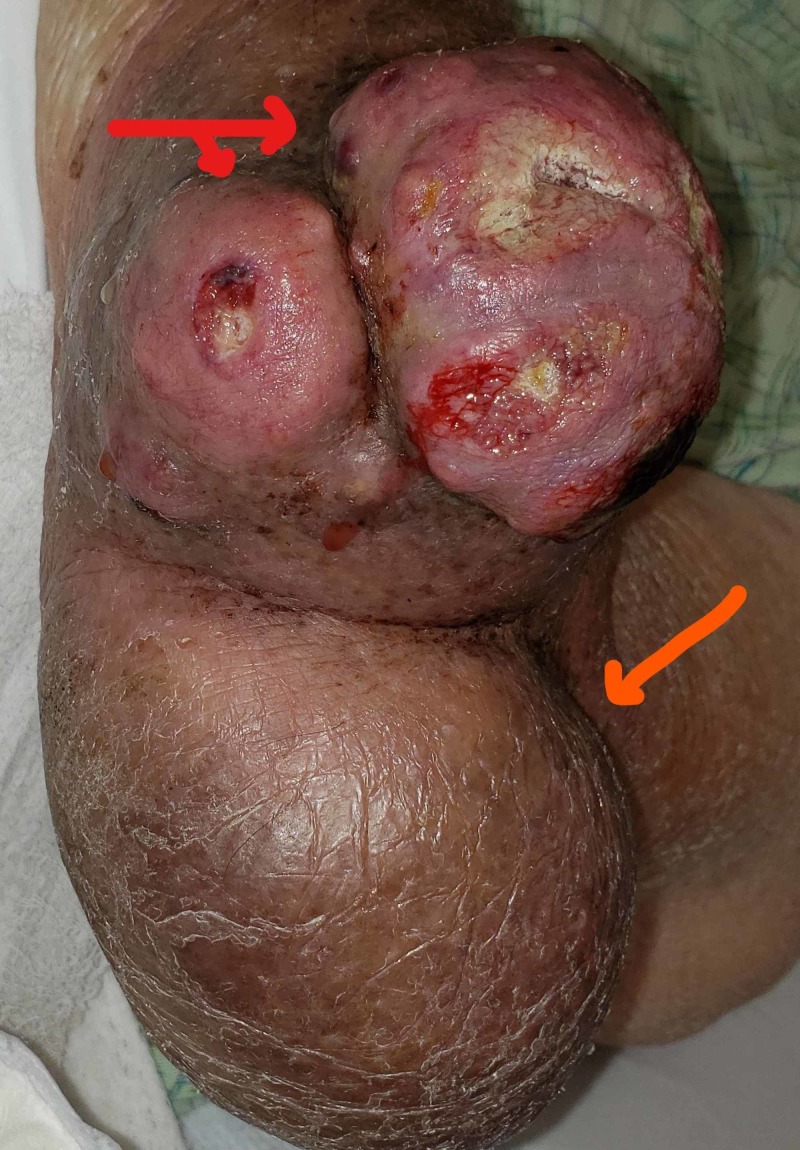
Two different masses over the left elbow Red arrow - new mass; orange arrow - gouty tophus present for 20 years

Physical examination showed a soft tissue mass of about 6x6 cm over the left elbow, just below the olecranon process, with a small area of skin necrosis and oozing of blood (Figure 1). The patient also had a large gouty tophus present for more than 20 years just proximal to the current mass (Figure 1). He was on chronic anticoagulation with coumadin for atrial fibrillation and found to have an elevated international normalized ratio (INR) of 3.0.

**Figure 2:**
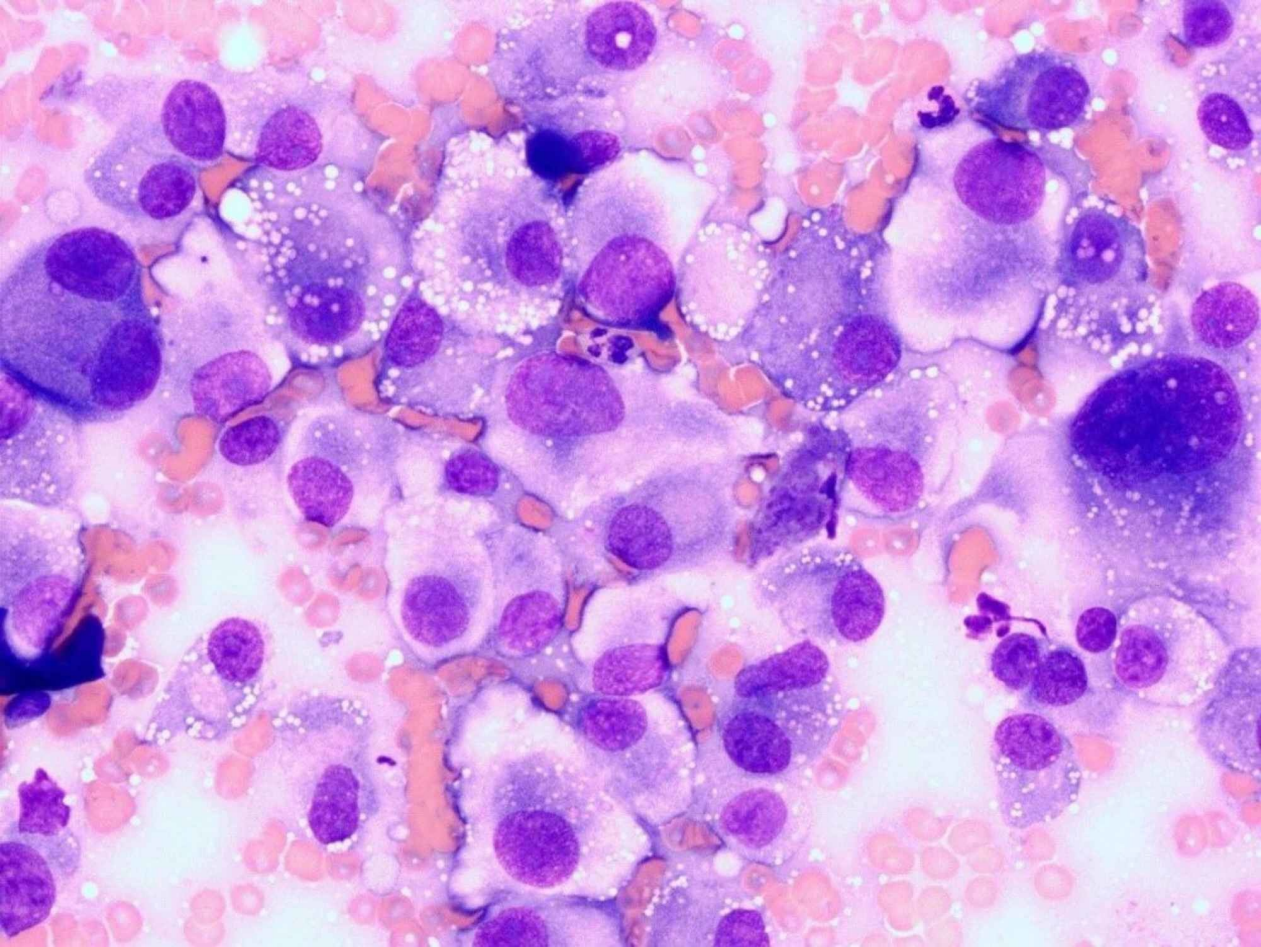
Atypical cells of histiocytic proliferation

The patient was admitted and treated for possible sepsis based on sepsis criteria. Coumadin was held and bleeding was stopped with a pressure dressing. Needle aspiration was done from the elbow mass with mild serosanguinous sterile fluid. Blood and fluid cultures were negative. Infectious diseases, orthopedic surgery, and rheumatology were consulted for the left elbow area mass. The patient had persistent leukocytosis (Table 1) even though he was clinically stable and the slowly growing mass for a couple of months raised suspicion for neoplastic disease. Dermatology was consulted, which did a bedside biopsy, with results favoring a histiocytic sarcoma (Figure 2). He underwent a bone marrow biopsy with no evidence of hemopoietic proliferative disorders.

**Table 1:** Persistent leukocytosis WBC: white blood cells

WBC	25.57 (H)	21.20 (H)	21.71 (H)	23.51 (H)	23.62 (H)	22.71 (H)	27.80 (H)	28.34 (H)	22.04 (H)	25.22 (H)	22.43 (H)	24.46 (H)	23.21 (H)	22.19 (H)	23.07 (H)	25.94 (H)	21.54 (H)	23.67 (H)	22.01 (H)	24.67 (H)
Hemoglobin	11.5 (L)	10.3 (L)	10.9 (L)	11.0 (L)	11.3 (L)	10.9 (L)	11.2 (L)	11.0 (L)	10.1 (L)	10.6 (L)	10.6 (L)	10.6 (L)	10.0 (L)	10.2 (L)	10.4 (L)	10.4 (L)	10.2 (L)	10.1 (L)	10.1 (L)	10.2 (L)
Hematocrit	37.7 (L)	33.5 (L)	35.2 (L)	34.9 (L)	36.5 (L)	35.7 (L)	36.6 (L)	36.5 (L)	33.4 (L)	34.6 (L)	33.4 (L)	34.7 (L)	33.5 (L)	32.8 (L)	32.8 (L)	33.3 (L)	32.6 (L)	32.8 (L)	32.7 (L)	31.8 (L)
Platelets	204	185	161	186	181	158	170	163	154	163	158	163	157	163	172	194	147 (L)	169	164	171

Due to his advanced age with multiple comorbidities, including decompensated heart failure, along with worsening renal failure complicated by severe protein-calorie malnutrition and deconditioning, the patient became severely deconditioned with declining quality of life. Considering his overall prognosis, the patient and family together opted for comfort measures only and the patient passed away with hospice.

## Discussion

Histiocytic sarcoma is a rare neoplasm of mature histiocytes, presents as a painless mass most commonly extranodal in origin, and can arise as a primary tumor or as a transformation of a pre-existing low-grade B-cell lymphoma. About 2% to 8% of chronic lymphocytic leukemia cases can transform into a histiocytic sarcoma [2]. Most cases were reported in adults with a slight male predominance [3].

The diagnosis of histiocytic sarcoma is made after an incisional/excisional biopsy based on the immunohistochemical delineation of the characteristic expression of one or more histiocytic markers. Specifically, the presence of macrophage-associated antigens, CD68, and lysozyme immunophenotypically confirm the histiocytic lineage. Also, CD163 has been recently characterized as a more specific marker for histiocytic neoplasms. In cases of histiocytic sarcoma arising from B-cell lymphomas, there may be a retention of some B-cell markers [4]. Typically, HS are negative for CD1a, CD21, and CD35, and they do not express B-cell, T-cell, or myeloid markers. The differential diagnosis includes other sarcomas such as rhabdomyosarcoma, Langerhans cell sarcoma, epithelioid sarcoma, and melanoma. Histologically, the tumor cells are usually large in size with abundant eosinophilic cytoplasm and eccentric round to oval nuclei. Small and distinct nucleoli with vesicular chromatin are present [2].

The standard of treatment is surgical with local resection. Adjuvant radiotherapy and chemotherapy are used especially for multifocal presentations. Bone marrow transplantation is an option depending on the presentation and immunophenotypes [5-6].

## Conclusions

Histiocytic sarcoma is an aggressive tumor with about 50% mortality. It is a rare epithelioid malignancy often misdiagnosed with other sarcomas and cases of non-Hodgkins lymphoma. Histiocytic sarcoma may arise primarily as a soft tissue tumor and tumor size may be a prognostic factor. Hence, high clinical suspicion and vigilant pathological examination with specific immunohistochemical phenotyping are essential and play an important role in early diagnosis and, possibly, prognosis.
